# The impact of behavioural screening on intervention outcomes in a randomised, controlled multiple behaviour intervention trial

**DOI:** 10.1186/1479-5868-8-24

**Published:** 2011-03-30

**Authors:** Lauren A Waters, Elisabeth A Winkler, Marina M Reeves, Brianna S Fjeldsoe, Elizabeth G Eakin

**Affiliations:** 1 The University of Queensland, School of Population Health, Cancer Prevention Research Centre, Brisbane, Queensland, Australia

## Abstract

**Background:**

With an increasing research focus on multiple health behaviour change interventions, a methodological issue requiring further investigation is whether or not to employ pre-trial behavioural screening to exclude participants who are achieving a pre-specified level of one or more behaviours. Behavioural screening can be used to direct limited resources to participants most in need of a behaviour change intervention; but may reduce the representativeness of the sample and limit comparability with trials that do not employ pre-trial behavioural screening. Furthermore, the impact of this type of screening on intervention participation and intervention effects is unknown.

**Methods:**

Data for this study come from the Logan Healthy Living Program, a randomised, controlled telephone counselling lifestyle intervention trial which did not employ behavioural screening prior to randomisation. Screening for physical activity, diet or the combination was simulated using baseline trial data. To examine the impact of behavioural screening on intervention participation (in terms of participant characteristics, intervention dose received and retention), characteristics of participants included an excluded under the various screening scenarios were compared. To examine the impact of behavioural screening on intervention effects, results from the main trial analysis were compared with results obtained from the same analyses performed separately for each of the screened groups.

**Results:**

Simulated pre-trial behavioural screening impacted minimally on intervention dose received and trial retention rate. Beyond the anticipated effect of reducing baseline levels of the behaviours being screened for, behavioural screening affected baseline levels of behaviours not targeted by screening, and participants' demographic and health-related characteristics. Behavioural screening impacted on intervention effects in ways that were anticipated and positive, but also unexpected and detrimental. Physical activity screening (alone or in combination with diet) resulted in improved intervention effects for physical activity, while fruit and vegetable screening had no impact on intervention effects for these outcomes. All three types of screening impacted detrimentally on intervention effects for behaviours not being targeted by screening.

**Conclusions:**

Behavioural screening may have desirable and undesirable consequences in the context of multiple behaviour intervention trials, and thus its potential merits and pitfalls should be carefully considered.

## Background

Participating in regular moderate intensity physical activity and consuming a healthy diet are fundamental to the prevention and management of many prevalent chronic diseases [1-4]. There is now a large body of literature addressing the development and evaluation of intervention trials that aim to increase participation in physical activity and encourage healthy nutrition. These behaviours have traditionally been targeted individually, but more recently, research has suggested that targeting multiple behaviours concurrently may maximise health outcomes [5].

The shift towards multiple health behaviour interventions brings with it new challenges related to trial design [6]. A particular methodological issue requiring further investigation is whether or not to employ behavioural screening prior to the commencement of the trial in order to exclude participants who are achieving a pre-specified level of the target behaviours. Behavioural screening is a relatively straightforward process in the context of single behaviour intervention trials. However, in multiple behaviour intervention trials, the complexity of behavioural screening (i.e., whether to screen on one, some, or all behaviours) is amplified, and the extent to which this practice influences trial participation and intervention effects is uncertain.

One rationale for employing pre-randomisation screening relates to the potentially beneficial impact this practice may have on intervention effect sizes. In theory, behavioural screening may favourably influence intervention effect sizes via several mechanisms: for example, by selecting a sample with a greater capacity for change and in which ceiling effects are minimised [7]. Alternatively, screening out participants who are already engaging in a particular behaviour may result in a sample that is potentially more resistant to change in the absence of an intervention [8], thereby potentially reducing the likelihood of control group improvements and their attenuation of intervention effects. Another reason for employing behavioural screening prior to the commencement of an intervention trial is that it can be used to direct limited resources to participants for whom behaviour change is a greater public health priority (i.e., those who are not currently achieving a pre-specified level of the target behaviours) [9]. The downside of such exclusions is that study samples may become less representative of the sampling frames from which they were drawn, reducing the generalisability of study findings.

Given that behavioural screening has the potential to influence intervention effects, the outcomes of studies that adopt different behavioural screening practices may not be comparable. A snapshot of the literature describing telephone-delivered intervention trials targeting physical activity and/or diet in adults illustrates that there is substantial variation in behavioural screening practices, across behaviours and between single versus multiple behaviour intervention trials. We examined all intervention trials identified in a recent systematic review [10], plus all literature published from January 2006 until June 2009 that matched this review's selection criteria. Screening for diet (alone or in combination with physical activity) occurred rarely in diet (2/8) and multiple behavior (2/8) intervention trials. By contrast, screening for physical activity (alone or in combination with diet) occurred in most (18/23) physical activity intervention trials but in less than half (3/8) of the multiple behavior intervention trials. The tools used to screen for behaviours, the specific aspects of the behaviors targeted by screening (particularly for diet), and cut-points used to determine eligibility (particularly for physical activity screening) varied greatly.

This study aimed to investigate the impact of screening for physical activity, diet, or the combination on intervention participation and intervention effects in the context of a multiple behaviour intervention trial. Data come from a recently completed randomised trial of a telephone-delivered physical activity and diet intervention that did not use behavioural screening [11]. To simulate pre-trial behavioural screening, participants were excluded according to baseline levels of physical activity and/or diet. The following specific research questions were posed:

1. Does behavioural screening impact on intervention participation in terms of the characteristics of participants recruited, intervention dose received and retention rates?

2. What is the impact of behavioural screening on intervention effects for all behavioural outcomes (both those screened for and those not)?

3. Where behavioural screening impacts on intervention effects, is this due to an effect on intervention group change, control group change, or both?

## Methods

The Logan Healthy Living Program (LHLP) was a 12-month, telephone-delivered lifestyle intervention trial promoting physical activity and diet among primary care patients with type 2 diabetes and/or hypertension in a socioeconomically disadvantaged community bordering Brisbane (the capital city of the state of Queensland), Australia. This intervention was evaluated in a cluster-randomised controlled trial conducted between February 2005 and November 2007, with data analysed between February and June 2009. The study protocol was approved by The University of Queensland, Human Research Ethics Committee. A detailed description of the methodology of this trial has been previously reported [12].

### Participant recruitment

Eligible participants were primary care patients with type 2 diabetes or hypertension, who were aged 30 years or older, with a telephone number and without contraindications to participation. The trial took a population-based approach and included all participants regardless of their dietary practices or physical activity levels prior to the trial (i.e., pre-trial behavioural screening was not conducted). This was done partly because the study aimed to gauge the effectiveness of the intervention on a representative sample of primary care patients, and partly because it was assumed that when targeting participants with chronic disease, the vast majority would display poor physical activity and/or diet relative to national recommendations. Additionally, active participants may still gain clinical benefit by further improvements in physical activity because the upper limit beyond which clinical improvement is unattainable is not known [4].

A recruitment flow chart and analysis of participation has been reported elsewhere [12]. In summary, the study had a high consent rate (n = 434, 72.6% of eligible contacts), minimal evidence of selection bias [12], high retention to end-of-intervention (78.6%), minimal differences between completers versus drop outs, and no difference in dropout between the study groups [11].

### Telephone Counselling Intervention

Details of the intervention, which was guided by social-cognitive theory [13], and used motivational interviewing techniques [14], have been described in detail elsewhere [12]. Briefly, participants from practices randomised to receive telephone counselling (TC) received up to 18 telephone calls and supplementary materials over a 12-month period. The number of calls completed for each participant was systematically tracked.

### Usual Care Group

Participants from practices randomly assigned to the usual care (UC) condition were mailed a 1-page letter with brief feedback on their behavioural results after each assessment. They also received quarterly project newsletters with general health tips and off-the-shelf brochures on a variety of health topics, including physical activity and diet.

### Outcome Measures

All study data were obtained using computer-assisted telephone interviews at baseline, four- and 12-months, by trained interviewers who were blind to study condition. Self-reported minutes per week of moderate-to-vigorous physical activity were assessed via the Active Australia Survey [15], which has been found to be reliable [16] and of acceptable validity [17] within Australian adults. Test-retest reliability, convergent validity and criterion validity of this survey are similar to those of the International Physical Activity Questionnaire (IPAQ) and the US Behavioral Risk Factor Surveillance Survey (BRFSS) [17].

Self-reported daily intake of fibre (grams per day), and total and saturated fat intake (as a percentage of total energy) were measured by the Anti-Cancer Council of Victoria Food Frequency Questionnaire, which estimates intakes of most nutrients accurately (within 10%) and does not systematically under- or overestimate against weighed records [18]. Servings of fruit and vegetables per day were measured using items from the Australian National Nutrition Survey [19], which have demonstrated validity against more comprehensive self-report measures and biomarkers, including serum carotenoids and red-cell folate [20].

### Behavioural screening

The primary outcomes of the intervention trial were related to diet and physical activity; therefore these are the behaviours for which screening was simulated. Three retrospectively screened samples were generated using baseline health behaviour data: those screened for physical activity, diet, or the combination. Physical activity screening excluded participants who met the national physical activity guidelines at baseline (i.e., ≥150 minutes of moderate-to-vigorous physical activity over 5 or more sessions per week) [21]. Diet screening (based on fruit and vegetable intake) excluded participants who met national recommendations for both fruit and vegetable intake at baseline (i.e., ≥2 servings of fruit and ≥5 servings of vegetables per day). Combined screening excluded participants meeting guidelines for physical activity *or *fruit and vegetable intake. Only a very small number of participants (n = 12) were physically active *and *consuming a healthy diet, so the effect of screening to exclude participants engaging in both behaviours was not examined.

### Statistical analysis

Under each behavioural screening scenario, included and excluded participants were compared in terms of their: demographic characteristics (age, gender, ethnicity, marital status, education, employment, household income); health-related characteristics (presence of diabetes and/or hypertension, number of self-reported chronic conditions, smoking, body mass index [BMI]); baseline levels of the behavioural outcomes; intervention dose received (TC participants only); and study retention. Comparisons were made using t-tests (normal, continuous variables), Mann-Whitney tests (non-normal, continuous variables) or chi-square tests (categorical variables). Differences ≥10% between the participants who were excluded and those who were included were reported as noteworthy, regardless of statistical significance.

To examine the impact of behavioural screening on intervention effects, the main analysis of our trial data [11] was compared with results obtained from the same analysis performed separately for each of the screened groups. To summarize, behavioural changes from baseline to 12 months were assessed, within the TC and UC groups, and between groups, on an intention to treat basis, with imputation of no change from baseline for participants who withdrew or were lost to follow-up. Three participants who died of unknown causes during the trial were excluded. Change scores (12-months minus baseline) for physical activity and diet (fruit, vegetable, fibre, total and saturated fat intake) were used as outcomes because these approximated a normal distribution. Linear mixed models (in SAS version 9.2) included the effects of group, time, group by time and baseline values of outcomes (to control regression to the mean) [22], and used random intercepts for participant and primary care practice in view of the repeated measures and cluster design. Results for between-groups intervention effects are reported as adjusted means with standard errors or 95% confidence intervals. A ≥10% relative difference in intervention effects in the screened samples compared to the non-screened sample is reported as a potentially meaningful impact of behavioural screening.

## Results

The original study sample included 434 participants. Using physical activity screening, 323 participants were retained (26% excluded); diet screening retained 394 participants (9% excluded); and combined screening retained 298 participants (31% excluded).

### Effect of behavioural screening on intervention participation

#### Characteristics of participants recruited

For all three behavioural screening criteria, there were no statistically significant differences between included and excluded participants with respect to age, gender, ethnicity, marital status, employment or household income (Table 1). Physical activity screening led to a significant, systematic exclusion of participants with higher levels of education (p = 0.014 for linear trend), and diet screening led to a noteworthy (≥10%), but not statistically significant difference for weekly household income, where participants earning at least $1500 AUD tended to be excluded.

**Table 1:** Baseline characteristics of participants - with and without screening for physical activity, diet, or both.

	All participants (unscreened)	Participants included with physical activity screening	Participants included with diet screening	Participants included with physical activity or diet screening
	**(n = 434)**	**(n = 324)**	**(n = 394)**	**(n = 299)**

*Intervention participation ^a^*				
Intervention dose (calls) received (median, interquartile range, n)	13 (8-16), n = 228	13 (8-16), n = 171	13 (8-16), n = 208	13 (8-16), n = 158
Retention rates, n(%)	341 (78.6)	254 (78.4)	309 (77.8)	230 (76.9)
*Health-related characteristics*				
Type 2 diabetes, n (%)	197 (45.4)	147 (45.4)	180 (45.3)	137 (45.8)
Hypertension, n (%)	371 (85.5)	280 (86.4)	340 (85.6)	257 (86.0)
Chronic conditions				
1-2 conditions, n (%)	175 (40.3)	125 (38.6)	158 (39.8)	115 (38.5)
3-4 conditions, n (%)	189 (43.5)	141 (43.5)	172 (43.3)	128 (42.8)
5+ conditions, n (%)	70 (16.1)	58 (17.9)	67 (16.9)	56 (18.7)
*p *^b^		*0.078*	*0.223*	*0.052*
Body mass index, kg	31.1 (6.8)	31.2 (7.2)	31.1 (6.9)	31.3 (7.2)
Current smoker, n (%)	60 (13.8)	46 (14.2)	56 (14.1)	44 (14.7)
*Demographic attributes*				
Age, years	58.2 (11.8)	58.4 (11.7)	58.1 (11.8)	58.3 (11.7)
Gender, n (%) Female	265 (61.1)	204 (63.0)	241 (60.7)	188 (62.9)
Ethnicity, n (%) Caucasian	395 (91.0)	294 (90.7)	362 (91.2)	270 (90.3)
Marital status, n (%) Married/living together	309 (71.2)	229 (70.7)	281 (70.8)	210 (70.2)
Education				
Primary or less	88 (20.3)	73 (22.5)	78 (19.6)	66 (22.1)
Junior high school	151 (34.8)	114 (35.2)	136 (34.3)	102 (34.1)
Senior High school	46 (10.6)	36 (11.1)	43 (10.8)	33 (11.0)
Trade or technical diploma	99 (22.8)	67 (20.7)	93 (23.4)	65 (21.7)
University Degree	50 (11.5)	34 (10.5)	47 (11.8)	33 (11.0)
*p for trend*		***0.014***	*0.118*	*0.248*
Employment, n (%) Retired	157 (36.2)	113 (34.9)	141 (35.5)	103 (34.4)
Weekly household income, n (%) $ ≥1500AUD	75 (20.2)	55 (19.6)	64 (19.0) ^Δ^	48 (18.8)
*Physical Activity*				
Physical activity (minutes/week) median (25^th^, 75^th ^percentile)	60 (0, 210)	0 (0, 70)***^c Δ^	60 (0, 210)	0 (0, 75)*** ^Δ^
Physical activity (sessions/week) median (25^th^, 75^th ^percentile)	2 (0, 2)	0 (0, 2) *** ^Δ^	2 (0, 2)	0 (0, 2)*** ^Δ^
*Diet*				
Total fat (% calories)	36.9 (5.2)	37.2 (5.2)*	37.0 (5.2)	37.3 (5.2)*
Saturated fat (% calories)	14.4 (3.3)	14.6 (3.3)**	14.4 (3.4)	14.7 (3.3)**
Fibre intake (grams/day)	21.8 (8.0)	21.8 (7.9)	21.3 (7.4)*** ^Δ^	21.3 (7.6)** ^Δ^
Vegetables (servings/day)				
median (25^th^, 75^th ^percentile)	3 (2, 4)	3 (2, 4)	3 (2, 4)*** ^Δ^	3 (2, 4)**
Fruit (servings/day)				
median (25^th^, 75^th ^percentile)	1 (1, 2)	1 (1, 2) ^Δ^	1 (1, 2)*** ^Δ^	1 (1, 2)*** ^Δ^

^a ^Data are given as mean (SD) or N (%) unless otherwise specified.

^b ^Linear-by-linear association chi-square

^c ^Bold cells represent behaviours predicted to be impacted by screening

^Δ ^Substantial difference (≥10%) between included and excluded participants

* p < 0.05 (included participants v excluded participants)

** p < 0.01

*** p < 0.001

Participants included and excluded under the various behavioural screening scenarios were not significantly or substantially different in terms of BMI, the proportion of current smokers, or the proportion diagnosed with hypertension or type 2 diabetes (Table 1). The difference in number of self-reported chronic conditions between participants included and excluded by combined screening approached statistical significance (chi square = 3.772, df = 1, p = 0.052).

As anticipated, behavioural screening effectively and significantly reduced baseline levels of behaviours being screened for (Table 1). While diet screening (based on fruit and vegetable intake) also resulted in the exclusion of participants with significantly higher baseline fibre intake, it had no impact on baseline levels of other dietary behaviours or on physical activity. Interestingly, physical activity and combined screening did affect baseline levels of behavioural characteristics not being screened for; with excluded participants having significantly lower total fat and saturated fat intake (as a percentage of total calories) than included participants. This difference was most marked under the physical activity screening scenario, where excluded participants had 1.4% lower total fat and 1.1% lower saturated fat intake (as a percentage of total calories) than included participants. The median number of fruit serves per day also tended to be higher (by one serve per day) in participants excluded with physical activity and combined screening, compared to those who were included, although the comparison was significant only for combined screening.

#### Intervention dose received

Mann-Whitney tests revealed that telephone counselling call completion, a measure of intervention dose, was not statistically or substantially different for included versus excluded TC group participants under the physical activity (p = 0.657), diet (p = 0.656), or combined (p = 0.105) screening scenarios. The median number of completed calls was 13 (of a possible 18) for the all screened groups and the unscreened sample.

#### Retention rates

Retention rates (Table 1) were not substantially or statistically different between participants who were included versus excluded with physical activity (chi square = 0.024, df = 1, p = 0.878); diet (chi square = 1.660, df = 1, p = 0.198); or, combined screening (chi square = 0.935, df = 1, p = 0.334).

### Anticipated consequences of behavioural screening

Table 2 presents the between-group intervention effects and within-groups changes observed in the unscreened sample, as published previously [11], and the three screened samples.

**Table 2:** Adjusted baseline to 12 months mean change^a ^in behavioural outcomes with and without behavioural screening

	All participants (unscreened)	Participants included with physical activity screening	Participants included with diet screening	Participants included with physical activity or diet screening
	**(n = 431)**	**(n = 324)**	**(n = 394)**	**(n = 299)**

*Physical activity (minutes/week)*				
Telephone Counselling (TC)	71.16 (14.28)***	113.15 (13.96)***^c^	62.03 (15.02)***	103.27 (14.35)***
Usual Care (UC)	84.48 (14.92)***	78.46 (14.71)***	86.00 (15.65)***	76.15 (15.14)***
TC - UC	-13.32 (-53.91, 27.26)	**34.70 (-5.22, 74.61) ^Δ^**	-23.96 (-66.60, 18.67) ^Δ^	**27.12 (-13.95, 68.19) ^Δ^**
*Fruit intake (servings/day)*				
TC	0.50 (0.06)***	0.49 (0.07)***	0.52 (0.06)***	0.49 (0.07)***
UC	0.20 (0.06)**	0.23 (0.07)**	0.23 (0.07)**	0.28 (0.08)**
TC - UC	0.30 (0.12, 0.47)	0.26 (0.07, 0.46) ^Δ^	**0.29 (0.10, 0.47)**	**0.21 (+0.00, 0.42) ^Δ^**
*Vegetable intake (servings/day)*				
TC	1.05 (0.24)***	0.95 (0.26)***	1.21 (0.23)***	1.13 (0.24)***
UC	0.34 (0.25)	0.25 (0.26)	**0.53 (0.23)**	**0.43 (0.25)**
TC - UC	0.71 (0.03, 1.39)	0.70 (-0.03, 1.39)	**0.68 (0.04, 1.32)**	**0.70 (0.02, 1.39)**

	(n = 426)^b^	(n = 320)^b^	(n = 389)^b^	(n = 295)^b^

*Total fat intake (% calories)*				
TC	-1.98 (0.25)***	-1.95 (0.35)***	-2.06 (0.30)***	-1.99 (0.34) ***
UC	-0.83 (0.31)**	-1.01 (0.36)**	-0.93 (0.31)**	-1.12 (0.36)**
TC - UC	-1.15 (-1.98, -0.32)	-0.94 (-1.93, 0.05) ^Δ^	-1.13 (-1.98, -0.28)	-0.87 (-1.84, 0.11) ^Δ^
*Saturated fat intake (% calories)*				
TC	-1.57 (0.25)***	-1.60 (0.27)***	-1.59 (0.26)***	-1.65 (0.28)***
UC	-0.60 (0.26)*	-0.66 (0.27)*	-0.63 (0.27)*	-0.70 (0.29)*
TC - UC	-0.97 (-1.68, -0.26)	-0.94 (-1.69, -0.19)	-0.97 (-1.71, -0.23)	-0.95 (-1.74, -0.16)
*Fibre intake (g/day)*				
TC	1.83 (0.46)***	1.52 (0.57)*	2.06 (0.48)***	1.74 (0.57)**
UC	-0.40 (0.48)	-0.63 (0.59)	0.19 (0.50)	-0.02 (0.60)
TC - UC	2.23 (0.93, 3.53)	2.15 (0.54, 3.77)	1.87 (0.50, 3.24) ^Δ^	1.76 (0.12, 3.40) ^Δ^

^a ^Adjusted means (standard error or 95% CI) of change from baseline to 12-months (adjusted for baseline values), with random intercepts for practice and subject.

^b ^Sample size differs due to the exclusion of participants with invalid data from the food frequency questionnaire.

^c ^Bold cells represent behaviours predicted to be impacted by screening

^Δ ^Substantial change (≥10%) in intervention effects when behavioural screening is employed compared to when it is not.

* p < 0.05 (for change from baseline)

** p < 0.01

*** p < 0.001

#### Impact of physical activity screening and combined screening on physical activity intervention effects

For the entire unscreened sample, there was no significant intervention effect (TC mean change minus UC mean change) on minutes per week of moderate-to-vigorous physical activity (-13 minutes), with large and significant changes occurring to a similar extent within both the TC and UC groups. Both physical activity and combined screening had a substantial impact on intervention effects for physical activity. Compared with when screening was not employed, intervention effects under the physical activity and combined screens more strongly favoured the TC group (+34 minutes and +27 minutes, respectively), although remained non-significant. Examining changes within groups under both these behavioural screening scenarios shows that the difference in intervention effects is primarily due to larger increases in physical activity being observed in the TC group when physical activity or combined screening was undertaken compared with when screening was not used; changes in physical activity were similar in the UC group regardless of behavioural screening.

#### Impact of fruit and vegetable screening and combined screening on fruit and vegetable intervention effects

In the unscreened sample, intervention effects for fruit and vegetable intake were significant, with intake increasing by +0.30 and +0.70 serves per day respectively. Diet screening had no impact on intervention effects for fruit or vegetable intake. Combined screening also had no impact on intervention effects for vegetable intake, but reduced the size of the intervention effect for fruit intake to +0.21 serves per day (a 30% reduction in the size of the intervention effect relative to the effect for the unscreened sample). Examining changes within groups showed that this detrimental impact on the intervention effect was primarily due to a comparatively larger increase in fruit intake being observed in the UC group when combined screening was employed than when behavioural screening was not employed.

### Unanticipated consequences of behavioural screening

#### Impact of physical activity screening on fruit and vegetable intake

The intervention effect for fruit intake under the physical activity screening scenario was substantially reduced (a 13% relative reduction). The intervention effect for vegetable intake under this behavioural screening scenario was not meaningfully affected, although it did lose statistical significance, possibly as a result of the smaller sample size.

#### Impact of fruit and vegetable screening on physical activity intervention effects

The intervention effect for physical activity was more strongly in favour of the UC group, when diet screening was employed, and this change appeared to be due to a reduced improvement in the TC group relative to when diet screening was not used.

#### Impact of all behavioural screening scenarios on intervention effects for variables not screened for (total and saturated fat [% daily calorie intake] and fibre intake)

With the exception of fibre intake, which may be correlated with fruit and vegetable intake, it was not expected that intervention effects for the dietary variables not targeted by behavioural screening would be impacted by the various screening scenarios. In comparison to the unscreened sample, intervention effects for fibre were reduced with diet screening (by 16%) and combined screening (by 21%), however the absolute magnitude of the differences in intervention effects were very small (0.4 grams or less).

Intervention effects for saturated fat were unaffected by screening. Physical activity and combined screening both impacted detrimentally on intervention effects for total fat intake. Compared with intervention effects for the unscreened sample, intervention effects for total fat intake were reduced by 18% and 24% respectively, under the physical activity and combined screening scenarios. In both cases, the impact on intervention effects appeared mostly due to larger reductions in total fat intake in the screened UC groups than the unscreened UC group.

## Discussion

To our knowledge, this is the first study to empirically evaluate the impact of behavioural screening on intervention participation or intervention effects within the context of a multiple behaviour intervention trial. Behavioural screening exerted an influence on intervention participation in terms of the characteristics of participants included in the sample, but had no impact on the intervention dose received or trial retention rate. Behavioural screening also impacted on intervention effects, in ways that were anticipated and positive, but also unexpected and detrimental.

The detrimental impact of behavioural screening on outcomes that were not screened for suggests that some intervention effects may have been unintentionally affected to a small extent in the minority of multiple behaviour intervention trials that have used behavioural screening. While, in the context of this study, the absolute magnitude of the detrimental impacts were often small, and of potentially limited clinical significance, the findings from this study suggest that the impact of screening in multiple behaviour intervention trials warrants further consideration. Our results intimate that screening for physical activity is likely to have the anticipated consequence of improving intervention effects for that behaviour, while diet screening based on fruit and vegetable intake, may not. Hence, the greater propensity to screen for physical activity in single rather than multiple behaviour intervention trials may lead to an overall underestimation of the capacity of the latter to demonstrate successful changes in physical activity.

Our findings also have implications for sample sizes required in future trials. Hypothesised intervention effects for physical activity should take into account whether the sample will be screened or not, with unscreened samples being likely to achieve smaller effect sizes and thus require markedly larger sample sizes. An alternative to using pre-trial screening to exclude certain participants is to use sub-group analyses to derive similar benefits in terms of effect sizes. However, particularly for lower prevalence behaviours (e.g., smoking), the required sample sizes may become prohibitively large.

Our study also highlights some potential mechanisms through which screening may exert both positive and detrimental influences. The benefits of physical activity and combined screening on intervention effects for physical activity were due to relatively greater improvements in TC group participants when these screens were employed than when screening was not used. Presumably, the findings suggest reduced ceiling effects (i.e., participants with lower baseline levels of physical activity had a greater capacity to improve in response to the intervention). For the other detrimental impacts, the mechanisms were less clear cut: sometimes changes in the TC group were affected, sometimes UC, sometimes both. Since retention and intervention dose were unaffected by screening, these can be ruled out as possible explanations for the impact on intervention effects. The impact could relate to the effect of screening on baseline levels, and possibly the impact on participant characteristics (as certain types of participants achieve different levels of success in behavioural trials) [23].

Behavioural screening reduced the baseline levels of the behaviours being screened for, consistent with its purpose of targeting participants who have the greatest need for a behavioural intervention [24]. However, physical activity and combined screening (unlike screening for diet only) also significantly and substantially affected baseline levels of behaviours not targeted by behavioural screening, specifically increasing baseline levels of total and saturated fat intake (as a percentage of daily calories). This is not entirely surprising as health behaviours (including physical activity and diet) tend to co-occur [25,26]; hence excluding participants with higher levels of physical activity is also likely to exclude those with healthier diets. Although our comparisons were limited by the small numbers of participants excluded, behavioural screening also tended to exclude participants with certain demographic and health-related characteristics, thus resulting in a less representative sample than was achieved without screening. Similarly, other studies have reported that adherence with recommended levels of multiple health behaviours is greater in samples with certain demographic characteristics [27]. The changes in the demographic profile of the sample occurring with screening resulted in the selection of a sample with characteristics associated with multiple unhealthy behaviours, who are therefore potentially in greater need of an intervention [28].

The results of this study must be interpreted in light of the fact that this is a secondary analysis, with 'behavioural screening' conducted retrospectively using baseline measures. In order to imitate feasible behavioural screening practices, we used behaviour-based questions and employed the national physical activity guidelines and fruit and vegetable intake recommendations as cut-off criteria. These criteria are not perceptibly different from those used by other studies, although our review of the literature did find that behavioural screening practices vary greatly in terms of both the specific aspects of the behaviours targeted (particularly for diet screening), and the criteria used to determine eligibility (particularly for physical activity screening). Despite our realistic simulation of behavioural screening, it is possible that true behavioural screening, occurring some time prior to baseline, and based on different, behavioural measures, may have had slightly different effects on intervention outcomes than what was found in our analyses. Both in our study and in general, the use of self-reported physical activity when screening is problematic, as over-reporting may lead to the exclusion of participants who would potentially benefit from the intervention.

A number of other issues must also be considered. As anticipated when targeting a group with chronic disease, behavioural screening excluded only a small number of participants due to the high prevalence of physical inactivity and unhealthy dietary practices in our study sample. If we had screened to exclude those with a health behaviour with a higher prevalence (e.g., non-smoking) or targeted a healthier population, we may have excluded a larger proportion of the sample and thus seen greater effects of behavioural screening [5,29]. For multiple behaviour trials that target a more diverse array of behaviours (including smoking, hazardous drinking and sun exposure) the complexity of screening is increased, and further research on the impact of screening for less prevalent behaviours is warranted. Also, our study was powered on the full sample (n = 434), so the loss of significance of intervention effects observed in the screened groups should not be over interpreted; changes to the size of effect should also be considered. Finally, the impact of screening may relate to the mechanisms previously discussed, but alternatively could be due in part to phenomena related to measurement error and biases of self report, as we used self-report measures of physical activity and diet that may be subject to over- or under-reporting [30,31]. For example, screening could have an impact on outcomes by tending to exclude participants who are comparatively more or less prone to socially desirable reporting. Without objective measures, this is not something we could assess.

## Conclusions

Behavioural screening is an inconsistent practice that may have greater appeal for some types of trials (e.g., efficacy trials targeting single behaviours only) than others (e.g., multiple behaviour intervention trials). Multiple behaviour intervention trials that target both diet and physical activity tend not to screen participants for pre-intervention levels of health behaviours. Based on our study findings, this appears to be good practice, but possibly one that makes it difficult to compare the results of single and multiple behaviour intervention trials. An alternative strategy for future multiple behaviour intervention trials is to conduct sub-group analyses, planned in advance of the trial, on an *a priori *defined sub-group of participants who are not attaining some pre-specified baseline level of the target behaviours. This approach could have the same benefits as behavioural screening (producing samples and results comparable to studies that screen) without the adverse consequences. Not screening also allows a more representative group to be recruited, enhancing generalisabiltiy and the ability to examine issues of effectiveness - which is important in informing the translation of research into practice [32].

## Competing interests

The authors declare that they have no competing interests.

## Authors' contributions

LW, EW, MR and EE were involved in the conceptualisation of the study. EW and LW conducted the data analysis and drafted the manuscript. MR also provided assistance in interpretation of the data and drafting of the manuscript. EE and BF provided assistance in drafting of the manuscript. All authors read and approved the final manuscript.
